# Defects in gut and enteric nervous system development in *Talpid3* mutant chicken

**DOI:** 10.1186/2046-2530-1-S1-P85

**Published:** 2012-11-16

**Authors:** JM Delalande, AM Campbell, N Thapar, AJ Burns

**Affiliations:** 1 UCL Institute of Child Health, UK

## Background

The *Talpid3* gene (KIAA0586) encodes a centrosomal protein essential for primary cilia formation and for normal Hedgehog (Hh) signalling. Since the Hh signalling pathway has been implicated in the development of the gastrointestinal tract and enteric nervous system (ENS), our aim was to examine *Talpid3* mutant embryos to gain further insight into the role of the Hh pathway in the development of these tissues.

## Methods

Fertilized eggs from the *Talpid3* flock (The Roslin Institute), were incubated for up to 8 days. Embryos were fixed at different time points, and processed for immunohistochemistry to identify cell types, and in situ hybridization for components of the shh pathway.

## Results

Macroscopically, the gut of *Talpid3* mutants was normally patterned but overall gut length was significantly reduced. Although ENS precursors and neurons were distributed along the length of the gut, ENS cells were scattered rather than arranged in typical plexuses. Also, the organisation of smooth muscle actin (SMA) and the patterning of the shh pathway components were considerably altered in mutants.

## Conclusions

We describe a number of phenotypic defects in *Talpid3* mutant gut. The reduced gut length suggests a lack of smooth muscle cell proliferation. The disorganized pattern of SMA and ENS suggests that cilia-mediated Hh signalling is essential for orchestrating the harmonious patterning of the different cell types necessary for normal gut development. We also demonstrate that the *Talpid3* chicken is an excellent model to study related human gut abnormalities, such as tracheoesophageal fistula and anorectal malformations, which are Hh-dependent.

